# TFEB; Beyond Its Role as an Autophagy and Lysosomes Regulator

**DOI:** 10.3390/cells11193153

**Published:** 2022-10-07

**Authors:** Berenice Franco-Juárez, Cristina Coronel-Cruz, Beatriz Hernández-Ochoa, Saúl Gómez-Manzo, Noemi Cárdenas-Rodríguez, Roberto Arreguin-Espinosa, Cindy Bandala, Luis Miguel Canseco-Ávila, Daniel Ortega-Cuellar

**Affiliations:** 1 Departamento de Neurodesarrollo y Fisiología, División de Neurociencias, Instituto de Fisiología Celular, UNAM, Mexico City 04510, Mexico; 2 Departamento de Biología Celular y Tisular, Facultad de Medicina, UNAM, Mexico City 04510, Mexico; 3 Laboratorio de Inmunoquímica, Hospital Infantil de México Federico Gómez, Secretaría de Salud, Mexico City 06720, Mexico; 4 Laboratorio de Bioquímica Genética, Instituto Nacional de Pediatría, Secretaría de Salud, Mexico City 04530, Mexico; 5 Laboratorio de Neurociencias, Instituto Nacional de Pediatría, Secretaría de Salud, Mexico City 04530, Mexico; 6 Escuela Superior de Medicina, Instituto Politécnico Nacional, Mexico City 11340, Mexico; 7 Departamento de Química de Biomacromoléculas, Instituto de Química, Universidad Nacional Autónoma de Mexico, Mexico City 04510, Mexico; 8 Neurociencias Básicas, Instituto Nacional de Rehabilitación Luis Guillermo Ibarra Ibarra, SSa, Mexico City 14389, Mexico; 9 Facultad de Ciencias Químicas, Campus IV, Universidad Autónoma de Chiapas, Tapachula Chiapas 30700, Mexico; 10 Laboratorio de Nutrición Experimental, Instituto Nacional de Pediatría, Secretaría de Salud, Mexico City 04530, Mexico

**Keywords:** transcriptional factor EB (TFEB), cellular senescence, DNA damage repair and cell cycle, WNT signaling, endoplasmic reticulum stress, carbohydrate, lipids, metabolism

## Abstract

Transcription factor EB (TFEB) is considered the master transcriptional regulator of autophagy and lysosomal biogenesis, which regulates target gene expression through binding to CLEAR motifs. TFEB dysregulation has been linked to the development of numerous pathological conditions; however, several other lines of evidence show that TFEB might be a point of convergence of diverse signaling pathways and might therefore modulate other important biological processes such as cellular senescence, DNA repair, ER stress, carbohydrates, and lipid metabolism and WNT signaling-related processes. The regulation of TFEB occurs predominantly at the post-translational level, including phosphorylation, acetylation, SUMOylating, PARsylation, and glycosylation. It is noteworthy that TFEB activation is context-dependent; therefore, its regulation is subjected to coordinated mechanisms that respond not only to nutrient fluctuations but also to stress cell programs to ensure proper cell homeostasis and organismal health. In this review, we provide updated insights into novel post-translational modifications that regulate TFEB activity and give an overview of TFEB beyond its widely known role in autophagy and the lysosomal pathway, thus opening the possibility of considering TFEB as a potential therapeutic target.

## 1. Introduction

The transcriptional factor EB (TFEB) belongs to the basic helix–loop–helix (bHLH) leucine zipper transcription factor, a member of the microphthalmia family (MiT/TFE), which includes microphthalmia-associated transcription factor (MITF), transcription factor E3 (TFE3), and transcription factor EC (TFEC). The members of the MiT/TFE family are proteins with a basic helix–loop–helix leucine zipper region that allows them to form homodimers or heterodimers between members of the same family of MiT/TFE transcription factors; the DNA regions recognized by MiT/TFE members are the Ephrussi boxes (E-box) formed by the sequence CANNTG. Specifically, TFEB recognizes E-boxes present on the promoter of autophagy genes. Moreover, E-box is present in the CLEAR motif (Coordinat-ed Lysosomal Expression and Regulation), a 10-base-pair motif (GTCACGTGAC) located in the promoter region of lysosomal genes [1,2,3]. As a protein that is capable of being imported or exported to the nucleus, TFEB possesses a nuclear localization signal (NLS) identified between amino acids 241 and 252, while the nuclear export signal (NES) is located at 140–150 amino acids of the human TFEB [4,5].

TFEB is considered a master regulator of autophagy and lysosomal function and is conserved through evolution [6,7,8]. TFEB also regulates another cellular process, which we will discuss later. TFEB increases autophagic flux by promoting the biogenesis of lysosomes, autophagosomes, and fusion with lysosomes, to efficiently degrade complex molecules [9,10,11,12]. TFEB activation depends on cellular nutrients and general cell status. Thus, under nutrient-rich conditions, TFEB is located in the cytoplasm under basal cellular conditions and translocates to the nucleus in response to starvation, lysosomal stress, pathogen infections, ER stress, and exercise to promote organismal homeostasis [9,12,13]. Once activated, TFEB directly binds to the promoter sequences to augment the expression of autophagy–lysosome-related genes, and its dysregulation of TFEB activity might contribute to the development of several diseases, including hepatic steatosis, neurodegenerative diseases, cancer, and inflammatory diseases [14,15,16,17,18,19,20]. Therefore, TFEB might serve as a potential therapeutic target for the treatment of human diseases.

## 2. Post-Translational Control of TFEB Activity

### 2.1. Phosphorylation

**Figure 1:**
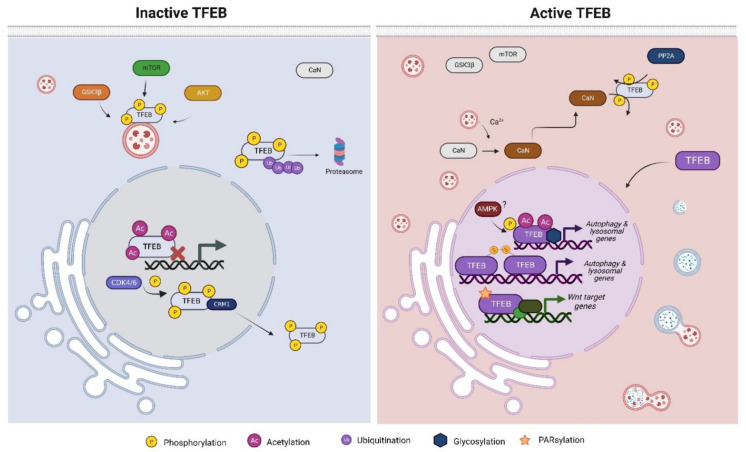
The TFEB activity is regulated by post-translational modifications. TFEB is mainly phosphorylated to avoid its nuclear translocation; however, depending on the cellular context, TFEB can be dephosphorylated, acetylated, glycosylated, or PARsylated.

The main function of TFEB known to date is the transcription of genes involved in diverse cellular processes. The activity of TFEB is dependent on cellular localization, which is controlled by translational modifications (phosphorylation, acetylation, etc.); thus, under cellular stress conditions such as starvation, DNA damage, or oxidative stress, TFEB may be located in the nucleus to promote its activity. Nevertheless, under basal conditions, phosphorylated TFEB lies inactive in the cytoplasm, where it is further degraded by the ubiquitin–proteasome pathway [21]. The phosphorylation of diverse serine residues is the main post-translational modification (PTM) that maintains TFEB in the cytoplasm; the main negative regulator of TFEB activity is the mammalian target of Rapamycin (mTorc1), which phosphorylates TFEB in at least three serines, S122, S142, and S211, although other kinases have been shown to promote TFEB phosphorylation (Figure 1) [13,22,23]. Glucose synthase kinase 3 beta (GSK3β) phosphorylates S134 and S138 to promote TFEB lysosomal surface localization together with small GTPases Rag, while serine-threonine kinase AKT (also named protein kinase B (PKB) has been shown to drive TFEB phosphorylation at S467 [24,25]. Although the phosphorylation of TFEB prevents its nuclear import, when the cellular context requires TFEB nuclear translocation, TFEB is dephosphorylated mainly by two phosphatases: Calcineurin and Protein Phosphatase 2 (PP2A) (Figure 1). Under starvation, the calcium stored in lysosomes effluxes, increasing cytosolic calcium concentration, thereby promoting the activation of CaN, a phosphatase activated by Ca^2+^, which removes phosphates from S142 and S211, favoring TFEB nuclear translocation [26]. PP2A is a phosphatase that is considered the master regulator of the cell cycle, as its dephosphorylate proteins are involved throughout cell cycle stages. Nevertheless, it has been demonstrated that under oxidative stress, PP2A dephosphorylates TFEB at several serine residues (S109, S114, and S122), including S211, the target serine of mTOR [27,28]. While starvation induces TFEB activation by inactivating mTOR and activating CaN, it has been demonstrated that TFEB can be activated independently of mTOR activity; this requires the removal of inactivating phosphorylated sites by phosphatases such as PP2A, suggesting that TFEB activity is subjected not only to one exclusive pathway but can be activated by the cell if a mechanism dependent on TFEB needs to be executed.

Although the cytoplasm retention of TFEB is well documented, and it occurs through phosphorylation events, once TFEB has been translocated to the nucleus and develops its transcriptional activity, it is exported outside by the exportin CRM1 (Chromosomal maintenance 1) (Figure 1) [29]. The first serine residue to be identified as necessary for nuclear export was S142, as it serves as a prime site for subsequent S138 phosphorylation by GSK3β. Moreover, further studies have revealed that residues S114, S142, T331, and S467 are phosphorylated by cyclin-dependent kinase 4/6 (CDK4/CDK6), although the critical residue that favors nuclear export seems to be S142 [30]. Phosphorylation appears to be the main PTM that prevents the activation of TFEB; however, Paquette et al. showed that once TFEB is translocated to nuclei, the AMP-activated protein kinase (AMPK) contributes to TFEB transcriptional activity as it phosphorylates TFEB at a serine cluster (S466, S467, and S469) (Figure 1). Although the AMPK target cluster is not indispensable for nuclear translocation, the phosphorylation of S466, S467, and S469 is necessary to sustain TFEB activity, as changes in the serine cluster by alanine prevent the transcription of TFEB target genes [31]. While some evidence suggests that the presence of a nuclear AMPK leads to an increase in nuclear TFEB, further studies need to be undertaken in order to elucidate whether TFEB is phosphorylated by nuclear or cytoplasmic AMPK [32]. To date, the phosphorylation status of TFEB seems to be the major mechanism that regulates its transcriptional activity; nevertheless, the activation of TFEB can be orchestrated, according to the cellular context, by other PTMs to ensure the proper function of TFEB.

### 2.2. Acetylation

TFEB acetylation has recently been proposed as a novel PTM that controls TFEB activity depending on the acetylated lysines (Figure 1). Acetylation at K116 blocks TFEB activity, as the substitution of K116Q (acetylation mimic) prevents degradation by the autophagy–lysosomal pathway of fibrillar amyloid-β (Aβ) by microglia [33]. Although there is no evidence that only the acetylation of K116 influences the subcellular localization of TFEB, acetylation at K116, together with K91, K103, and K430, are important for nuclear translocation and transcriptional activity, as mutations of lysine (K) to arginine (R) (deacetylated mimic mutation) decrease nuclear abundance, concomitant with autophagy flux reduction [34]. Other reports suggest that TFEB acetylation by the general control of amino acid synthesis 5 (CGN5) impairs the dimerization of TFEB and its binding to target promoter regions when TFEB is acetylated at K116, K274, and K279 [35]. Conversely, it was observed that the cytosolic deacetylase histone deacetylase 6 (HDAC6) could deacetylate TFEB and limit its activity by sequestering in the cytosol, suggesting that the TFEB acetylation pattern affects its transcriptional activation and that there exists a regulatory relationship between HDAC6 and TFEB [36]. However, whether the acetylation of TFEB promotes its transcriptional activity or not is still a matter of debate, but it seems to be a novel mechanism that might influence its activity.

### 2.3. Other Post-Translational Modifications of TFEB

Beyond phosphorylation and acetylation, TFEB has been shown to be modified by SUMOylating, PARsylation, glycosylation, and cysteine oxidation. The addition of small ubiquitin-like modifiers (SUMO) at the lysine present in the consensus sequences ΨKXE occurs on members of the MiT/TFE family; although TFEB preserves one of the consensus sequences, and SUMOylating arises at K316, we have yet to determine the effect of SUMOylation on TFEB activity and the cellular context that could mediate the addition of SUMO on TFEB [37].

Poly-ADP-ribosylation (PARsylation) is a reversible PTM executed by poly (ADP-ribose) polymerases (PARPs) that mediates the transfer of ADP-ribose from Nicotinamide adenine dinucleotide (NAD+) to the acceptor proteins [38]. Tankyrase 1 (TNKS1), also known as (PARP5A), is a PARP that PARsylate TFEB when the WNT/β-catenin signaling pathway is activated; intriguingly, the PARsylation of TFEB leads to the formation of a trimeric complex with the coactivator β-catenin and the transcription factors TCF1/LCF1 to induce the transcription of WNT target genes (Figure 1) [39,40]. To date, TFEB PARsylation is the only PTM that prevents the canonical transcription of TFEB target genes, suggesting that TFEB activity is not subjected exclusively to the transcription of autophagy and lysosomal genes.

TFEB glycosylation has been documented to occur during host infection by *Legionella pneumophila* as a mechanism to ensure nutrient availability by activating catabolic pathways such as autophagy. SetA is a glucosyltransferase from *L. pneumophila* that directly glycosylates TFEB at S138 and hampers S138 phosphorylation by GSK3β; therefore, NES is disrupted, and TFEB is not exported outside the nucleus (Figure 1). Likewise, glycosylation at S195 or S196, T201 or S203, and T208 disrupts the binding of 14-3-3 probably by masking the phosphorylation site of a target mTOR site, the serine 211 [41]. It is worth noting that, until now, the glycosylation of TFEB has not been shown to be mediated by the glucosyl–transferase property of the cell and has been proven to occur only during the infection of *L. pneumophila*; nevertheless, it might be possible that under specific cell circumstances, TFEB could be a target of glycosylation, which would promote its nuclear retention.

The oxidation of cysteine (Cys) residues by reactive oxygen species (ROS), specifically by hydrogen peroxide (H_2_O_2_), has been considered a mechanism that controls intracellular signaling cascades, as Cys oxidation promotes the formation of disulfide bonds with thiol groups, thereby changing the protein function [42]. Although Cys residues are not very abundant on proteins, they are present in functional regions. Interestingly, Martina et al. [43] showed that TFEB contains a Cys residue at position 212 of human TFEB, which is oxidized and favors the formation of oligomers with other TFEB molecules (Figure 1). The formation of oligomers prevents the deactivation of TFEB by mTOR, presumably by preventing the access of the kinase to its target sites [43]. The presence of many post-translational modifications over TFEB highlights the importance of the fine regulation of its transcriptional activity, allowing the cell to execute the mechanisms that define its cellular fate.

## 3. TFEB on Senescence

Senescence is a form of stable cell cycle arrest that prevents the proliferation of cells with unresolved or accumulated damage, such as telomere attrition, mitochondrial dysfunction, DNA damage, perturbed proteostasis, loss of nuclear integrity, oncogene activation, or autophagy atrophy [44,45]. Although the cell has stopped proliferating, it remains viable and metabolically active as senescent cells secrete a plethora of cytokines, chemokines, matrix metalloproteinases (MMPs), and growth factors, collectively known as senescence-associated secretory phenotype (SASP). It is noteworthy that SASP secretion can induce senescence in a paracrine fashion in neighboring cells [46,47]. Cellular senescence is distinguished by the expression of antiproliferative proteins (p16INK4A and p21CIP1), increased senescence-associated β-galactosidase activity, the persistent activation of DNA damage response (expression of histone family member X gamma and γ-H2AX), the stabilization of lamin A/C, and the decreased expression of lamin B, among other things [46,48,49]. The induction of senescence and the concomitant secretion of SASP can play beneficial roles when senescence is transient, as it contributes to embryonic development and wound healing and limits tumor progression; however, the accumulation of senescent cells can contribute to tissue dysfunction, leading to the development of age-related pathologies such as arthritis, neurodegeneration, diabetes mellitus, and cancer progression [50,51,52,53,54,55].

**Figure 2:**
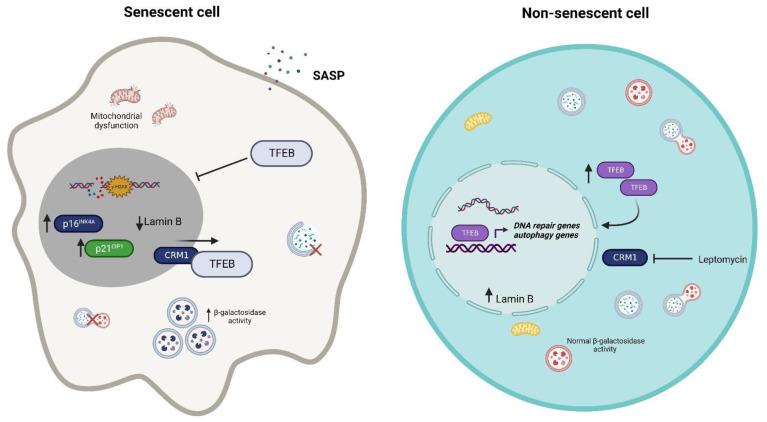
The TFEB activation can influence the induction of senescence. Decreased nuclear TFEB localization may be one of many causes of senescence; however, restoring TFEB nuclear activity could contribute to avoiding cellular senescence, perhaps through the induction of autophagy or DNA repair genes.

Dysfunctional autophagy has been considered a cellular senescence inducer as damaged proteins or organelles cannot be removed; in line with this, the knockdown of autophagy genes ATG7 and ATG5 in the human primary fibroblast leads to the increased expression of p16INK4A and p21CIP1 and increases β-galactosidase activity. Interestingly, the knockdown of ATG genes induces the accumulation of damaged mitochondria and increases ROS production [56]. The induction of senescence after H_2_O_2_ treatment in NIH3T3 cells impairs autophagic flux and decreases lysosomal activity. Nonetheless, the activation of AMPK, a kinase that activates autophagy, restores autophagy flux and lysosomal activity and prevents senescence development [57]; however, autophagy activation has also been shown to promote senescence. As such, the relationship between autophagy and senescence might need further investigation [58]. TFEB, as the main controller of autophagy and lysosome biogenesis, has been linked to senescence in both in vivo and in vitro models of intervertebral disc degeneration, where TFEB decreases its nuclear abundance, leads to lysosomal dysfunction, and decreases autophagy flux. As a consequence, β-galactosidase activity increases, and the expressions of p16INK4A and markers of SASP, such as interleukin-6 (IL-6) and matrix metalloproteinase 3 (MMP3), increase as well. However, when TFEB is overexpressed, senescence is not induced, and autophagy flux is restored (Figure 2) [59]. The accumulation of senescent cells in aged organisms is considered one of many contributors to aging [60]. In the brains of mice at 18 months, specifically in the hippocampus and cortex, Wang et al. [61] showed the decreased expression of nuclear TFEB compared with mice aged 6 months, as well as the increased expression of p16INK4A and γ-H2AX; however, they also found that the overexpression of TFEB ameliorated senescence markers and improved learning and memory skills in mice of the same age [61]. Similar findings were made by Gorostieta et al. [62] in young and old mice hippocampus and cortex; notably, they found that the decreased nuclear TFEB was due to the increased expression of CRM1, which exports TFEB from the nucleus, and this was correlated with the expression of senescence markers in old mice. Interestingly, when they inhibited CRM1 in vitro with leptomycin, nuclear TFEB was augmented, and β-galactosidase activity was reduced; nevertheless, no changes were observed in lamin A/C and lamin B expression after TFEB nuclear restoration [62]. In a cellular model of chronic obstructive pulmonary disease (COPD) emphysema, β-galactosidase activity dropped when cells were treated with gemfibrozil to stimulate TFEB activity, suggesting that TFEB activation could prevent senescence induction. Nevertheless, additional senescence markers need to be outlined in order to propose senescence evasion by the activation of TFEB [63]. Although autophagy induction after TFEB overexpression or activation has been shown as the main mechanism of evading senescence, we cannot discard additional operating mechanisms that TFEB can orchestrate independently of autophagy to delay senescence. As we will discuss in the following section, TFEB induces the transcription of genes related to DNA repair; as the induction of senescence is the result of unresolved DNA damage, overexpressing TFEB might contribute to a better cell response after a cell stress insult and therefore contribute to the evasion of senescence.

## 4. TFEB on DNA Damage Repair and Cell Cycle

DNA integrity is essential to maintaining cellular and organismal health; therefore, mechanisms that ensure the proper repair and elimination of DNA damage under determined circumstances need to be provided. The DNA damage response (DDR) is a well-described pathway that senses and responds to different types of DNA damage [64].

p53, considered the “Guardian of the Genome”, acts as an integrator of many programs that define the fate of the cell according to the nature, intensity, and duration of stress, leading to cell cycle arrest or the activation of cell death programs [65]. For example, when DNA damage occurs during the G1 phase, p53 is stabilized, leading to the transcription of P21, an inhibitor of CDK4,6/cyclin-D, CDK2/cyclin-E, and CDK2/cyclin-A activity, thereby arresting the cell cycle [66,67]. Interestingly, TFEB has been recently proposed as a transcriptional amplifier in response to DNA damage through the activation and stabilization of p53 [68]. Brady et al. [68] showed that the induction of DNA damage with etoposide increased the nuclear translocation of TFEB, causing mTOR activity to decrease; interestingly, TFEB activation is p53-dependent, as the p53 knockout of mouse embryonic fibroblasts (MEFs) failed to activate TFEB. Intriguingly, the knockout of TFEB downregulates the expression of DDR genes after exposure to etoposides, including the p53 transcript. Moreover, p53 is less stable when TFEB is not expressed, suggesting that p53 and TFEB might regulate each other in response to DNA damage. The reduced levels of p53 might be due to the increased levels of MDM2, an E3 ubiquitin ligase that renders p53 for proteasome degradation; nevertheless, the increased levels of MDM2 seem not to be TFEB-dependent, and importantly, overexpressing the active TFEB increases the stability of p53 as its half-life is extended. Brady et al. [68] also proposed TFEB as a mediator of cell fate after DNA damage, as its expression is necessary for apoptosis cell death. Similar findings were made by Slade et al. [69] in relation to breast cancer cells, where TFEB is activated and favors the transcription of DNA repair genes, specifically the homologous recombination genes, thereby increasing the DNA damage repair capacity of breast cancer cells. Remarkably, the activity of the phosphatase calcineurin is necessary for TFEB activation, but the induction of TFEB activity results in the evasion of apoptosis cell death [69].

p21, a target of p53, can be regulated positively by TFEB [70]. Analyses of the promoter of p21 found one motif region recognized by TFEB. Moreover, the chromatin immunoprecipitation of TFEB on HeLa cells stably expressing active TFEB demonstrates the binding of TFEB to the promoter region of p21. Interestingly, the knockout or knockdown of TFEB suggests p21CIP1 downregulation, indicating a TFEB requirement; nonetheless, the transcription of p21 after DNA damage is not exclusively due to TFEB, as its expression still requires p53 [70]. Contrary to treatment with etoposide, where mTOR activity was reduced, and therefore TFEB was translocated to the nucleus, doxorubicin treatment did not lead to mTOR inhibition, suggesting that TFEB activation is independent of mTOR [70]. Notably, the depletion of TFEB and treatment of cells with doxorubicin increase the number of cells at the G2-phase of the cell cycle. Nonetheless, the overexpression of TFEB and the concomitant increase in p21 CIP1 lead to an increased population of cells at the G1-phase, suggesting that, in the absence of TFEB and p21 after DNA damage, cells are arrested at the G2-phase [70]. The work of Brady and colleagues also shows the relevance of TFEB in the expression of cell cycle genes, specifically the genes involved in cell cycle checkpoints [68]; interestingly, further reports made by Doronzo et al. [71] show that, in endothelial cells (ECs), TFEB drives the expression of genes related to cell cycle, cell division, G1/S transition, and DNA replication, among other genes; notably, when TFEB is silenced, ECs halts proliferation at the G1-S cycle transition; interestingly, one of the direct targets of TFEB is the cyclin-dependent kinase 4 (CDK4), important for G1-S transition of the cell cycle [71]. Similar results were further reported in TFEB-depleted hepatoblasts, in which the authors showed a block of the G1-S cycle transition [72]. Together, the recent evidence shows the important role of TFEB in cell cycle control and progression.

## 5. TFEB on WNT Signaling

WNT/β-catenin signaling is an evolutionarily conserved pathway that controls cell proliferation, differentiation, and cell fate determination during embryogenesis and adulthood tissue homeostasis [73]. The control of WNT pathway activity is well documented, and two defined mechanisms control its activity: the WNT canonical (β-catenin-dependent) pathway and the noncanonical pathway (β-catenin-independent activity) [74]. The canonical pathway is initiated by the binding of WNT ligands to Frizzled receptors present on the cell surface. Then, β-catenin disassembles from the destruction complex containing Axin, adenomatous polyposis coli (APC), and GSK3β and accumulates; afterward, β-catenin is translocated to the nucleus and, as a transcriptional coactivator, it binds to and modulates the activity of the T-cell factor/lymphoid enhancer factor (TCF/LEF) transcription factors [73,74,75]. Interestingly, the activation of the WNT/β-catenin pathway with Wnt3a-conditioned media induces the nuclear translocation of TFEB independently of the phosphorylation status of TFEB, and surprisingly, nuclear TFEB after WNT/β-catenin stimulation does not transcribe lysosomal genes but promotes the transcription of WNT target genes [40]. Kim et al. [40] found the first evidence of the importance of TFEB PARsylation to the formation of a complex between TFEB and transcription factors of WNT signaling as a mechanism to promote the expression of WNT genes. That said, previous reports made by Li et al. [76] had found similar results regarding the relevant role of TFEB in activating TCF/LEF1 on gastric cancer cells to enhance migration and invasiveness, suggesting a positive loop within the TFEB and WNT/β-catenin signaling pathway, as WNT inhibitors decrease the protein stability of TFEB [76]. WNT signaling has been found to be dysregulated in many types of cancer; for example, mutations on regulatory proteins such as APC prevent the ubiquitination and subsequent degradation of β-catenin, leading to the sustained transcription of WNT target genes in colorectal cancer cells. Interestingly, previous reports made by Liang et al. [77] gave evidence on the role of TFEB in cancer progression, as TFEB promotes cell proliferation and migration; however, autophagy induction as a consequence of TFEB activation was suggested to be the main mechanism for tumor growth and metastasis [75,77]. In this context, recent studies indicate the involvement of TFEB in the control of cell proliferation and cell motility in endometrial, lung, pancreatic, prostate, and endothelial cancer cells [78,79,80,81,82]. The recent findings open the possibility of further research into how TFEB can contribute to the activity and regulation of additional pathways not related to autophagy.

## 6. Endoplasmic Reticulum Stress and TFEB

The endoplasmic reticulum (ER) is a metabolic organelle responsible for the synthesis of proteins, lipids, carbohydrates, and calcium storage. The ER is highly sensitive to cellular changes, and its homeostasis may be disrupted by several insults, including an imbalance between the rate of protein synthesis and folding capacity, dysregulation in calcium homeostasis, oxidative stress, and redox imbalance in the ER lumen. Perturbations in the functions of the ER provoke the accumulation of unfolded proteins and trigger ER stress, which in several contexts might lead to apoptosis when the response is sustained for a long time [83,84]. ER stress activates an adaptive signaling pathway called the unfolded protein response (UPR), which transduces signals related to the protein folding status of the ER lumen to the nucleus in order to trigger the expression of specific transcription factors to cope with stress conditions. Thus, UPR plays an important role in the pathogenesis of various metabolic diseases, such as diabetes, obesity, cancer, infectious diseases, and inflammatory diseases, among others [85,86,87]. Therefore, UPR components offer interesting targets for therapeutic intervention that may reduce stress levels and offer therapeutic benefits that improve human health [88].

**Figure 3:**
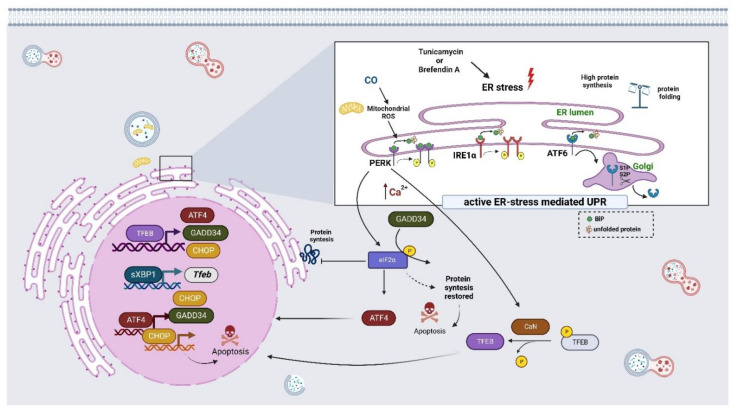
The TFEB regulates the expression of several regulators of the ER stress response. ER stress, triggered by several compounds, promotes the nuclear localization of TFEB in a PERK-, ATF6-, IRE1α-, and calcineurin-dependent manner that consequently induces the transcription of UPR-related genes to restore ER homeostasis and support cell survival or apoptosis, if the stress is persistent.

UPR signaling is initiated by the activation of three distinct types of stress sensors located at the ER membrane, including the protein kinase RNA-like endoplasmic reticulum kinase (PERK), the inositol-requiring enzyme 1 (IRE1), and the activating transcription factor 6 (ATF6) [89,90]. Briefly, the three UPR transmembrane sensors are normally bound to the ER-resident chaperone GRP78/BiP (glucose-regulated protein, 78kDa/Binding immunoglobulin Protein) to form a stable complex with the luminal domain, keeping them in an inactive monomeric state. However, under conditions of ER stress, GRP78/BiP is released from the UPR sensors and binds to the accumulated unfolded proteins, thus activating the signaling pathways mediated by PERK, IRE1α, and ATF6 [89]. After GRP78/BiP is dissociated from PERK, it becomes activated and oligomerized, phosphorylates itself, and in turn, phosphorylates the α-subunit of eukaryotic initiation factor 2α (eIF2α) at serine 51, and then halts translation by preventing ribosome assembly. This reduces the overload of proteins entering the ER of a stressed cell, which leads to attenuation in protein synthesis. In summary, this pathway inhibits mRNA translation, reduces the flux of proteins in ER, and ameliorates ER stress [91]. On the other hand, the low expression of eIF2α activates the transcription factor ATF4, which induces cell survival by increasing the expression of genes related to oxidative stress and amino acid synthesis [92]. However, if ER stress persists, ATF4 induces the transcription of a pro-apoptotic transcription factor CHOP (C/EBP homologous protein), which may trigger apoptosis. In addition, CHOP promotes the expression of GADD34 (growth-arrest and DNA damage-inducible 34), which dephosphorylates eIF2α, reinitiating translation and sensitizing cells to apoptotic signals [83,93]. IRE1α is a bi-functional transmembrane kinase/endoribonuclease that, in response to UPR, is activated, dimerized, trans-autophosphorylated, and undergoes a conformational change that activates its RNase domain. This, in turn, cleaves the intron of mRNA to XBP1 (X-box binding protein) in sXBP1, an active transcription factor that promotes the expression of genes associated with the UPR. XBP1s regulate the expression of genes encoding factors that modulate protein folding, secretion, ER-associated degradation (ERAD), and protein translocation into the ER [94,95]. ATF6 is also an ER transmembrane protein that contains a bZIP transcription factor on its cytosolic domain. Upon the excessive loading of misfolded proteins, ATF6 is sent to the Golgi apparatus for further processing and activation, where the proteases S1P and S2P release the N-terminal cytosolic portion that is translocated further to the nucleus, where ATF6 binds to ER stress response elements and stimulates the transcription of a subset of UPR target genes, such as DNA damage-inducible transcript 3 (DDIT3), chaperones BiP, and GRP94 (glucose-regulated protein 94) [93,96]. ATF6 exhibits cross talk with XBP1s by forming heterodimers, which may drive specific gene expression programs to reach ER proteostasis [97]. As previously mentioned, the cellular localization of TFEB is dependent on the cellular context; once in the nucleus, TFEB triggers changes in gene expression to cope with cellular stress. Initially, Martina et al. [98] showed that TFEB participates in UPR signaling since the induction of ER stress with tunicamycin or brefeldin A promotes the nuclear localization of TFEB in a PERK- and calcineurin-dependent fashion. Interestingly, the activation of TFEB induces not only the transcription of lysosomal- and autophagy-related genes but also those related to ER homeostasis and apoptosis, such as the transcription factor 4 (ATF4) and its targets genes (CHOP and GADD34), which are key deciding factors that integrate the UPR and integrated stress response. Thus, under ER stress conditions, the TFEB-mediated activation of ATF4 seems to occur via its direct binding to the CLEAR element in the promoter region of ATF4 in order to restore ER homeostasis and support cell survival (Figure 3) [98].

It has been shown that carbon monoxide (CO) may protect cells against ER stress via the activation of the PERK-dependent pathway [99]. The treatment of hepatic or HeLa cells with CO is able to promote TFEB nuclear translocation. In fact, a mild increase in mitochondrial ROS levels in response to CO treatment leads to PERK activation, Ca^2+^ release, and calcineurin-dependent TFEB nuclear translocation, which, in turn, promotes mitochondrial homeostasis through mitophagy and mitochondrial biogenesis (Figure 3) [100]. Similar findings emerged with the PERK activator (SB202190) in neuroblastoma cells, where activated PERK led to an increase in cytosolic Ca^2+^ levels that subsequently promoted the translocation of TEFB into the nucleus via the calcineurin-dependent dephosphorylation of TFEB. These findings suggest that the activation of PERK–TFEB signaling could decrease amyloidogenesis in neurodegenerative diseases [101]. It is well documented that UPR increases ER cross talk with autophagy in each of the three canonical branches of the UPR, and it activates autophagy, which permits the restoration of ER homeostasis. Recent work by Zhang et al. [102] showed that the promoter of *Tfeb* possesses a consensus sequence recognized by the transcription factor sXBP1. Interestingly, a ChIP assay showed the enrichment of sXBP1 on the promoter of *Tfeb* in primary hepatocytes treated with thapsigargin and in the livers of fasted mice. Moreover, the overexpression of sXBP1 increases the nuclear localization of TFEB and induces autophagy flux; together, the data demonstrated that the ER and autophagy are functionally coupled at the transcriptional level via the XBP1-mediated activation of TFEB (Figure 3) [102]. Similar findings were made when several cell line cultures or rodent models were treated with distinct compounds such as Kazinol C, Isobacachalcone, DFS, or Guanabenz, respectively; these were able to activate ER stress-associated UPR components such as PERK, ATF6, IRE1α phosphorylation, BiP, CHOP, ATG7, GADD34, and sXBP1, as well as the nuclear translocation of TFEB and autophagy. However, the underlying molecular details remain unknown [103,104,105,106].

The transforming growth factor-beta 1 (TGF-β1) significantly promotes protein synthesis, causing an excessive demand for the protein folding capacity of ER, resulting in ER stress and the concomitant activation of three branches (PERK, IRE1α, and ATF6) of UPR. Similar to other reports, here, the activation of UPR led to dependent TFEB autophagy induction. However, TFEB activation induces fibroblast differentiation and collagen secretion, processes that are linked to the formation of hypertrophic skin scars [107]. Similar findings have been reported in the osteoblast since TFEB may regulate its differentiation through the ATF4/CHOP signaling pathway in response to ER stress [108]. Together, the reports suggest that TFEB might ameliorate ER stress but can also influence cell differentiation together with the UPR signaling pathway.

## 7. TFEB and Its Influence on the Metabolism

In general, all living things are exposed to constant changes in nutrient supply in their life activities; therefore, to reach metabolic homeostasis, they must undergo the constant adjustment of internal metabolic activities in response to nutritional states. Energy metabolism is regulated through several aspects of cellular machinery that catalyze chemical reactions in order to generate energy as ATP that is consumed by anabolic reactions and supplied to organelles; this involves the conversion of simple chemical compounds into complex ones that are essential for cell growth. Some components involved in this regulation include enzymes and transcriptional factors that play an important role in this process. Relatively recent works suggest that TFEB could influence mammalian metabolism to maintain energy balance.

### 7.1. Role in Glucose Metabolism

Glucose homeostasis is critical to human health due to its central importance as a source of energy. For utilization, glucose is transported by members of the facilitative glucose transporter (SLC2) and by the sodium–glucose cotransporter (SGLT) family [109]. Glucose is metabolized via glycolysis and provides the energy molecule ATP and several metabolites for other metabolic pathways.

It has been reported that in the skeletal muscles of mice overexpressing TFEB might control several genes related to glucose metabolism. Specifically, TFEB overexpression is correlated with an increase in the expression of two genes for glucose transport (GLUT1 and GLUT4), genes for glucose phosphorylation (hexokinase 1 and 2), and an increase in glucose uptake. Interestingly, these genes contain a CLEAR sequence, suggesting that their regulation is TFEB-dependent and highlighting the importance of TFEB in the regulation of glucose homeostasis in skeletal muscle [110]. Consistent with these findings, the treatment of tumor-associated macrophages (TAMs) with chloroquine, an antimalarial drug, promotes lysosomal calcium release that contributes to TFEB activation and increases the presence of genes involved in glycolysis, such as GLUT1 and GLUT4, phosphofructokinase (PFKM), and pyruvate kinase (PKLR); these effects are TFEB-dependent since the regulation was blocked after TFEB knockdown. Collectively, these data suggest that TFEB reprograms the macrophage metabolism, from oxidative phosphorylation to glycolysis [111].

**Figure 4:**
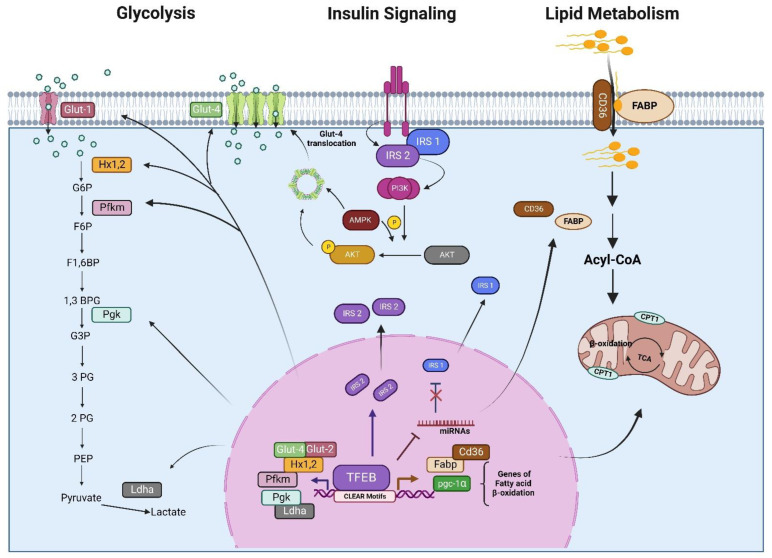
The TFEB regulates genes involved in carbohydrate metabolism, lipid metabolism, and insulin signaling. Once active, TFEB may promote energy utilization through the activation of several related genes. Some of the proposed metabolic processes regulated by TFEB are shown, together with the relevant substrates.

Strikingly, and contrary to the above-described findings, the genetic loss of TFEB (TFEB^−/−^) in murine cardiomyocytes led to an enrichment of genes related to glycolysis and gluconeogenesis, such as phosphofructokinase (Pfkp), glycerol-3-phosphate dehydrogenase (Gpd1), glucose 6-phosphate translocase (slc37a4), hexokinase 1 (hk1), and phosphoglucomutase (Pgm5), suggesting that glucose oxidation processes are dampened in TFEB^−/−^ cardiomyocytes [4]. In general, cancer cells require a key regulator to induce metabolic reprogramming, which sustains their cell growth. An analysis of the role of TFEB in metabolic processes in lymphoma cells found that its activation significantly reduces basal levels of glycolysis, glucose uptake, and some of its encoding genes, such as lactate dehydrogenase (Ldha) and phosphoglycerate kinase (Pgk), as well as the metabolite 2-phosphoglycerate (Figure 4). Conversely, there was an accumulation of glyceraldehyde 3-phosphate and glucose-6-phosphate that was consistent with reduced glycolytic flux [112]. These findings suggest that the effect of TFEB on the regulation of glucose metabolism might be tissue-dependent.

Insulin-activated Akt signaling in vascular endothelial cells (ECs) is involved in the regulation of systemic glucose metabolism and vascular homeostasis [113]. In ECs, insulin signaling starts with the binding of the hormone to the insulin receptor (IR) and the activation of insulin receptor substrate (IRS1, 2) by phosphorylation, which follows the activation of the PI3-kinase/Akt/eNOS pathway, where protein kinase B (AKT) phosphorylates the endothelial nitric oxide synthase (eNOS) at serine 1177, resulting in increased nitric oxide production and vasodilation [114]. In recent work, Sun et al. [115] studied the role of endothelial TFEB in glucose metabolism. The results showed that endothelial TFEB improves systemic glucose tolerance in mice via the activation of Akt-IRS1, 2. In fact, the overexpression of endothelial TFEB is related to upregulation at the transcriptional level of IRS2, which consequently augments the amount of IRS protein via the downregulation of several microRNAs (mirR-335-3p, miR-495-3p, and miR-548o-3p), leading to the activation of Akt signaling and glucose uptake through GLUT1 in ECs (Figure 4) [115]. Whether this mechanism of TFEB occurs in other cell types warrants future investigation. Interestingly, it has also been shown that the overexpression of TFEB reverses the suppressed glucose uptake of palmitate-induced insulin resistance in C2C12 myotubes via the restoration of the insulin-dependent GLUT4 signaling pathway and the activation of AMPK, a key energy sensor, which regulates glucose metabolism and has been shown to protect against insulin resistance. Therefore, it is proposed that TFEB acts as a critical regulator of glucose homeostasis in skeletal muscle cells [116].

### 7.2. Role in Lipid Metabolism

Fatty acids are molecules that can be obtained from dietary intake and de novo synthesis. Their catabolism generates small molecules that modulate several cell signaling pathways and play a critical role in transcriptional regulation [117]. Initially, Settembre et al. [118] showed that inducing starvation or overexpressing TFEB in the liver upregulates genes that encode for lipid metabolism, such as the transport of fatty acid chains across the plasma membrane (Cd36 and Fabps) for the oxidation of free fatty acids (FFA) in mitochondria (Cpt1, Crat, Acadl, Acads, and Hdad) and peroxisomes (Cyp4a) (Figure 4). Similarly, TFEB overexpression in inguinal white adipose tissue (iWAT) leads to a clear induction of adipose tissue browning-related genes, including uncoupling protein 1 (Ucp1), which encodes a key thermogenic protein, and mitochondrial and lipid metabolism genes, such as cell death-inducing DFFA-like effector a (CIDEA), aconitase 2 (ACO2), carnitine palmitoyltransferase 1B (CPT1B), cytochrome c oxidase subunit 5B (COX5B), and citrate synthase (CS). It is noteworthy that most of the effects of TFEB on lipid metabolism seem to be mediated by the direct regulation exerted by TFEB on the regulators of fat metabolism, such as the peroxisome proliferator-activated receptor-gamma coactivator (PGC1α) and peroxisome proliferator-activated receptor (PPARα) because, without PGC-α, the abilities of TFEB overexpression to drive TFEB-induced browning and to elicit beneficial metabolic effects were blunted [118,119]. Additionally, TFEB overexpression can rescue the obese phenotype and the glucose intolerance in knockout mice of the transcription factor E3 (TFE3), another member of the MiT family helix–loop–helix leucine zipper, suggesting that these transcription factors work together and cooperatively control the energy metabolism [120]. These effects appear to be evolutionarily conserved since HLH-30, the Caenorhabditis elegans homolog to TFEB, has been shown to activate the transcription of lipid catabolism genes and promote fat utilization upon food withdrawal [121,122]. Conversely, the genetic loss of TFEB^−/−^ in cardiomyocytes has different effects on lipid-related genes; specifically, the genes required for processing lipid intermediates (cholesterol, phospholipid, and sphingolipid) such as 2,4-dienoyl-CoA reductase 2 (Decr2), acyl-CoA synthetase family member 2 (Acsf2), ceramide-1-phosphate transfer protein (Cptp), lipase a (Lipa), StAR-related lipid transfer domain containing 3 (Stard3), sphingosine-1-phosphate lyase-1 (Sgpl1), and apolipoprotein E (ApoE) were upregulated, whereas those for lipid transport and metabolism genes, such as the cluster of differentiation 36 (CD36) and angiopoietin Like 3 (Angptl3), were downregulated [4].

**Figure 5:**
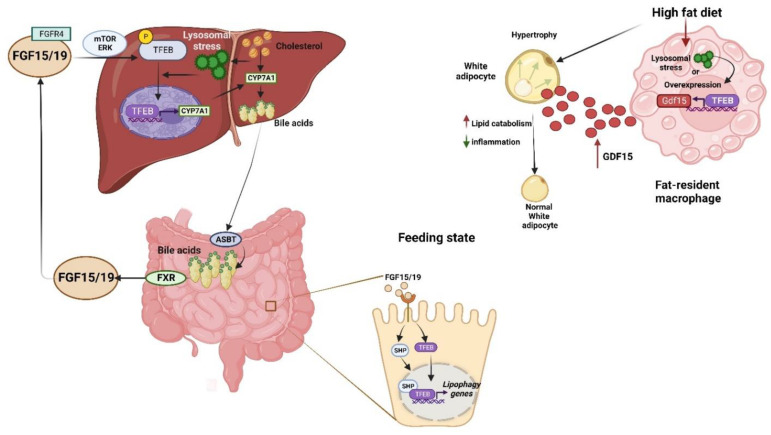
The TFEB is expressed in several tissues, including the liver, intestine, and white adipose tissue. In these cell types, in response to several stimuli, TFEB is activated and induces the expression of several genes, as indicated in the figure. In the liver, cholesterol accumulation causes lysosomal stress, which consequently induces TFEB nuclear translocation, activating the expression of cholesterol 7α-hydroxylase (CYP7A1) to produce hepatic bile acid synthesis that, in the intestine cells, promotes the production of the hormone fibroblast growth factor-15/19 (FGF15/19), and this consequently activates both the activate orphan nuclear receptor, the small heterodimer partner (SHP/NR0B2) and TFEB, to activates genes (Ulk1 and Atgl) essential for lipophagy. TFEB activation by overexpression or the occurrence of lysosomal stress in fat-resident macrophages protects against diet-induced weight gain and adiposity through the induction of growth differentiation factor 15 (GDF15)-enhanced adipose lipid catabolism, and it reduces inflammation.

It has been shown that TFEB can be dysregulated in lipid-related diseases. In nonalcoholic fatty liver disease (NAFLD) patients, hepatic steatosis alters the subcellular location of TFEB, being mainly cytosolic; as TFEB promotes genes related to lipid metabolism, TFEB activation could be an important factor in determining the severity of hepatic steatosis in NAFLD patients and might conceivably be related to reduced lipophagy activity [123]. Additionally, TFEB has been identified as an inductor of hepatic bile acid synthesis; in fact, bile acid synthesis is a major mechanism for preventing intrahepatic cholesterol accumulation and hypercholesterolemia. Wang et al. [124] found that excessive intracellular cholesterol accumulation causes lysosomal stress and subsequent TFEB nuclear translocation. TFEB activation induces cholesterol 7α-hydroxylase (CYP7A1) to promote bile acid synthesis, which promotes cholesterol catabolism and elimination (Figure 5). In addition, bile acids activate FXR to induce intestinal FGF15/19 and, thus, feedback, and they inhibit TFEB by causing TFEB phosphorylation and cytosolic retention (Figure 5) [124]. Furthermore, recent work shows that genetic loss of TFEB causes the reduction in the total cellular cholesterol in ECs, suggesting that TFEB is involved in cholesterol synthesis via the transcriptional modulation of genes encoding the sterol regulatory element-binding protein (SREBP-2), a key regulator of cholesterol, the SREBP cleavage-activating protein sterol sensor (SCAP), and the SREBP-2 target gene HMGCR (β-Hydroxy β-methylglutaryl-CoA reductase). Therefore, TFEB promotes the transcription of genes that drive the synthesis of cholesterol [82].

Generally, it is accepted that autophagy is activated under nutrient deprivation conditions and repressed in feeding conditions. Intriguingly, recent data show that after feeding, autophagy/lipophagy is activated in the intestine via the orphan nuclear receptor, the small heterodimer partner (SHP/NR0B2), the gut hormone, fibroblast growth factor-15/19 (FGF15/19), and the TFEB axis (Figure 5). After feeding, FGF15/19 is activated, and in turn, the nuclear localization of SHP and TFEB is increased via PKC-mediated phosphorylation, which consequently activates the transcription of genes (Ulk1 and Atgl) that are essential for lipophagy. This activation may reduce postprandial lipids via lipophagy and may provide novel targets for treating obesity-related metabolic disorders [125]. Intriguingly, TFEB expression was increased in macrophages of white adipose tissue (WAT) from obese mice and humans, whereas macrophage TFEB overexpression protects against obesity and insulin resistance. The livers of mice with HFD-induced weight gain and lipid accumulation were markedly reduced, probably because of the increased expressions of the fatty acid oxidation-related gene and a decrease in lipogenesis-related gene expression. Moreover, macrophage TFEB protected against HFD-induced obesity by inducing growth differentiation factor 15 (GDF15), suggesting that the activation of the TFEB–GDF15 axis could play a crucial role in regulating obesity-induced metabolic diseases (Figure 5) [126]. The estrogen-related receptor α (ERRα) drives lipid metabolism, together with PGC1α/PPAR; nevertheless, recent evidence shows that ERRα is a direct target of TFEB, and both are overexpressed in patients with endometrial cancer [41]. Intriguingly, TFEB and ERRα favor the invasion and metastasis of endometrial cancer cells by modulating lipid metabolism and increasing unsaturated fatty acid phosphatidylcholine, phosphatidylglycerol, and the ratio phosphatidylcholine/sphingomyelin, which together enhance membrane fluidity via epithelial–mesenchymal transformation signaling and promote EC progression [78].

## 8. Conclusions and Future Perspectives

Accumulated evidence has underscored the role of TFEB as a master regulator of lysosome biogenesis and autophagy. TFEB activation positively regulates the expression of autophagy and lysosomal biogenesis-related genes that promote the clearance of intracellular substrates through lysosomal exocytosis. However, increasing evidence suggests the crucial role of the TFEB protein in the control of several other vital cellular processes, such as cellular senescence, DNA repair, ER stress, carbohydrates metabolism, lipid metabolism, and WNT signaling-related processes that position TFEB as a key regulator responding to a variety of environmental cues. The newly identified noncanonical roles of TFEB have revealed how it is able to integrate multiple upstream stimuli to mediate specific downstream responses that conceivably could have medical relevance. Therefore, pharmacological strategies for TFEB activation or mitigation might be a promising approach in specific disease conditions, as is the case of metabolic or neurodegenerative diseases, and may hold relevance for a larger number of human diseases.
